# Gender Differences in Kidney Stone Disease (KSD): Findings from a Systematic Review

**DOI:** 10.1007/s11934-021-01066-6

**Published:** 2021-10-08

**Authors:** Kathryn Gillams, Patrick Juliebø-Jones, Siri Øvereng Juliebø, Bhaskar K. Somani

**Affiliations:** 1 Department of Urology, Great Western Hospital Swindon, Swindon, UK; 2 Department of Urology, Haukeland University Hospital, Bergen, Norway; 3 Department of Gynaecology, Telemark County Hospital, Skien, Norway; 4 Department of Urology, University Hospital Southampton, Southampton, UK; 5 Institue of Clinical Medicine (K1), University of Bergen, Norway

**Keywords:** Kidney calculi, Ureteroscopy, Percutaneous nephrolithotomy, Gender, Quality of life

## Abstract

**Purpose of Review:**

The prevalence of kidney stones is rising and historically carries a preponderance for males. However, recent evidence has questioned whether this gender gap is closing. The aim of this systematic review was to examine this further as well as evaluate possible underlying causes.

**Recent Findings:**

Recent evidence confirms the disparity in kidney stone disease between genders is closing. In the modern era, the rise in prevalence among females has been greater, especially in adolescence. Quality of life is also more adversely affected by kidney stone events among females who are also more likely to develop sepsis after endourological surgery. Males, however, are more likely to present with stone events during periods of high ambient temperatures

**Summary:**

Recent literature demonstrates a temporal change in the disease burden of KSD among men and women. The latter, especially adolescent females, are more likely to develop KSD in their lifetime compared to previous eras. Determining causation is complex and continued research is warranted.

## Introduction

Kidney stone disease (KSD) has a lifetime prevalence of 14%, and longitudinal studies have confirmed it is rising. Since the 1990s, the number of people affected by KSD has risen from 1 in 20 persons to 1 in 11 persons [1]. Given that 50% of patients will experience stone recurrence within 5 years, the resultant burden of KSD is extensive [2]. The associated cost impact on a global scale is estimated to exceed $4 billion by 2030 [3]. Saigal et al. determined the average number of working days lost per annum as a result of KSD is 19 days [4]. Cost analysis performed by Geraghty et al. revealed the cost of KSD is comparable to the combined cost of prostate cancer and bladder cancer in the UK [5].

In addition to the economic burden, the sequelae of KSD include deterioration in quality of life related to anxiety, fatigue and loss of sleep. A number of aetiological determinants are associated with KSD, and these include comorbid conditions such as hypertension and diabetes mellitus (DM) as well as lifestyle factors such as obesity and smoking [6, 7]. Understanding such causative factors can enable the clinician to drive tailored management and prevention strategies. While men are nearly twice as likely to develop KSD in their lifetime compared to women, the prevalence of KSD among the latter sex is increasing. The historical gender gap in KSD is therefore closing. Being able to curate advice further based on patient’s gender would heighten this personalized stone approach even further [8]. While an increasing number of original studies have been performed in an attempt to better understand the relationship between gender and KSD, evaluation and dissemination of these findings remains underreported. An understanding and appreciation of such evidence is of paramount importance to the clinicians as they strive to deliver the best care possible. This systematic review serves as an overview of these new findings based on the latest updates in the world literature.

## Methods

A systematic review of world literature was performed to identify original studies in English language, including basic science studies relating to KSD and gender differences. This was performed in accordance with the Preferred Reporting of Systematic Reviews and Meta-analysis guidelines (PRISMA) guidelines [9]. A time restriction of the past 5 years was placed in order to gain the most up to date findings. Search terms included (but not limited to) ‘male’, ‘female’, ‘gender’, ‘sex’, ‘urolithiasis’, ‘calculi’ and ‘stone disease’. Databases searched included Medline, EMBASE, Google Scholar and SCOPUS. Areas of interest that were identified and studied on gender differences included.

1. Epidemiology of the gender gap
2. Quality of life (QoL)
3. Role of hormones
4. Stone composition
5. Post intervention sepsis
6. Gender difference in Paediatrics
7. KSD and ambient temperature

## Results

From a total of 681 articles, 31 articles satisfied our pre-determined criteria and were selected for inclusion in our final review (Table 1).

**Table 1:** Summary of included studies with key findings from it

Author/year	Level of evidence	Sample size	Key finding
Gender gap			
Abufaraj/2020 [10]	IIb	34,749	Increasing numbers of previous pregnancies, menopause and female hormone use increases the risk of stone formation
Vaughan/2019 [16]	IIb	3364	Men significantly more likely to have symptomatic recurrence
Hsi/2018 [13]	IIb	42,136	Prevalence of KSD among female has risen
Masterson/2017	IIb	667,840	Incidence of KSD higher among female navy personnel
Tundo/2018 [11]	IIb	16,658	Women of working age were just as likely as men to develop KSD
Chen/2019 [12]	IIb	NR	Prevalence of KSD among female has risen
Kittanamongkolchai/2018 [15]	IIb	NR	Women are more likely to be asymptomatic stone formers
Quality of life			
Stern/2019 [18]	IIb	2052	Quality of life in women is more negatively impacted by stones than in men
Macraith/2020 [19]	III	142	Young women most likely to be re-admitted to ED post-URS
Patel/2017 [20]	III	103	Women under 40 are most adversely affected by KSD on quality of life
Islamoglu/2019 [21]	III	145	Young women most likely to experience SWL-related pain
Role of hormones			
Nackeeran/2020 [24]	IIb	10,193	No correlation between abnormal sex hormone levels and KSD
Peerapen/2019 [22]	III	–	In canine renal tissue, oestrogen reduces expression of calcium oxalate receptors, reduces crystal-binding capacity and reduces intracellular ATP
Zhu/2019 [23]	III	–	In rodent models, oestrogen deprivation led to increased calcium oxalate deposition in renal tissue and increased urinary oxalate excretion
Xiao/2020 [26]	IIb	7257	Female diabetics more protected from KSD by ACE inhibitors than male diabetics
Stone composition			
Kravdal/2019 [27]	IIb	1252	Men more likely to have calcium oxalate monohydrate stones; women more likely to form carbonate apatite and struvite stones
Wang/2020 [28]	IIb	1532	Male stone formers younger and more likely to have metabolic disorders; female stone formers more likely to have mixed stone composition and a history of UTI
Wood/2019 [29]	IIb	589	Male stone formers excrete more calcium and oxalate into their urine and have lower urinary pH and higher rates of urine supersaturation with uric acid
Post-intervention sepsis			
Southern/2019 [32]	IIb	3298	Women are more likely to develop sepsis after ureteroscopy, and more likely to have positive pre-operative urine cultures and infection stones
Lorenzo Soriano/2019 [33]	IIb	203	Women are more likely to develop infectious complications after PCNL than men
Díaz Pérez/2019 [35]	III	246	Women are more likely to develop urosepsis after ureteroscopy than men
Nevo/2017 [34]	IIb	1256	Female gender is a risk factor for post URS sepsis
Baboudijan/2020 [36]	III	604	Female gender is a risk factor for post URS sepsis
Özsoy/2015 [37]	III	927	Female gender is a risk factor for post URS sepsis
Wood/2020 [38]	IIb	227	Female gender is a risk factor for post URS sepsis
Paediatrics			
Schwaderer/2019 [43]	III	136	Girls with stones were more likely to have a positive leukocyte esterase test, were significantly shorter and less likely to have biochemical evidence of volume depletion than their male counterparts
Taisan/2016 [42]	IIb	152,925	Lifetime risk of developing KSD among females increased by 45% during study period
Meiouiet/2019 [44]	III	432	Majority of girls had calcium oxalate stones
Seasonal variation			
Vicedo-Cabrera/2020 [45]	IIb	132,597	Men are disproportionately affected by increased ambient temperature than women
Ordon/2016 [47]	IIb	423,396	Men most likely to present with KSD in high ambient temperatures
Fukuhara/2016 [48]	III	491	Men most likely to present with KSD in high ambient temperatures

### Epidemiology of Gender Gap

Seven studies have been reported since 2015, which offer up-to-date epidemiological perspectives on gender differences in KSD [10–16]. Abufraj et al. evaluated data from the US National Health and Nutrition Examination Survey (NHNES), and this revealed that between 2008 and 2018, the prevalence of KSD increased significantly among women (6.5% vs. 9.3%, (*p*_trend_ = 0.001) [10]. However, there was no significant increase among men. Risk factors specific to women were identified and included increasing number of previous pregnancies (2 pregnancies — OR 1.64, CI 1.1–2.23; 3 or more pregnancies — OR 2.17, CI 1.63–2.88), menopause (OR 1.61, CI 1.21–2.15) and female hormone use (OR 1.38, CI 1.15–1.65). Two further groups reported on the same national database [11, 12]. Tundo et al. focused their analysis on adults younger than 50 years of age in order to better understand the burden of the disease on working age group [12]. The authors determined that women of working age were just as likely as men to develop KSD. All the analysis carried out by the separate groups consistently reported that amongst adolescent females, the rate of increase was the greatest. Hsi et al. performed analysis of data from a prospective sample, the South Community Cohort Study, which also has confirmed the rise in female KSD [13].

In another retrospective review, Masterson et al. looked at over half a million Navy personnel and found the incidence of KSD in women was in fact higher than men (OR 1.17, *p* < 0.0001) [14]. Given the strict medical work up required in this area of work, to ensure safe flight status, this analysis included a large number of asymptomatic stones. It has been postulated that this may be one of the explanations why the rising trend is seen in the female population. Indeed, recent follow up data made available from the Olmsted County study reveals that women were more likely to be asymptomatic stone formers [15]. Vaughan et al. reviewed nearly 5000 stone episodes at a US centre and found that compared to women, men were significantly more likely to have a symptomatic recurrence following their initial presentation with stones (HR 1.25, *p* = 0.002) [16].

### Quality of Life

As part of the evolution for patient centred and holistic care, an increasing number of studies now include quality of life (QoL) as an outcome measure [17]. Four recent studies were identified, which explored this theme [18–21]. Stern et al. evaluated data from the North American Stone Quality of Life Consortium and found that younger, stone patients tolerated stone events less favourably than men [18]. Younger women had a significantly lower health-related quality of life (HRQoL) scores compared to older men (OR 1.56, *p* = 0.0003). Moreover, this difference was consistent across all four domains measured (social functioning, stone related impact, vitality and emotional functioning). This would be consistent with recent data from a study by Maccraith et al., which recorded that this group was most likely to have a re-admission to the emergency department after ureteroscopy (URS) [19]. Patel et al. carried out a survey to explore the impact of KSD on QoL and recorded a worsening trend in females under 40 years of age [20]. Previous evidence has shown that KSD impacts more negatively on care givers than primary income earners, and some author groups have speculated that this may offer some explanation to the above findings [18]. Islamoglu evaluated the role of menopause on pain experienced during shockwave lithotripsy (SWL) and reported women post menopause to have significantly lower pain scores than younger women (*p* < 0.001) [21].

### Role of Hormones

It has been hypothesised that sex hormones may have a significant role in gender differences for KSD, and 5 studies were identified which investigated this further [22–24, 26]. Peerapen et al. investigated the effects of oestrogen in the kidney [22]. They demonstrated that oestrogen (i) reduces surface expression of two calcium oxalate crystal receptors, (ii) reduces crystal binding capability and (iii) reduces intracellular ATP in renal tubular cells. These may all contribute to a favourable environment within the kidney, thus reducing the risk of stone formation in women of reproductive age. Zhu et al. used rodent models to demonstrate that oestrogen receptor knock-out mice and rats treated with an oestrogen receptor antagonist had increased renal calcium oxalate deposition and increased urinary oxalate excretion, and as a result, the activity of oestrogen in healthy females may be protective against KSD [23].

Conversely, Nackeeran et al. examined the possible role of sex hormones on the risk of stone formation by measuring the testosterone and oestrogen levels in over 10,000 American patients who self-reported a history of KSD [24]. They concluded that abnormal testosterone levels (both high and low) did not significantly increase the risk of stone formation. They also found no correlation between low oestrogen (in both genders) and KSD. However, given the timings of the study, the sex hormone levels were likely measured after some duration of the stone episode, so does not necessarily represent the levels at the time of stone formation.

There is some evidence to support a protective role of ACE-inhibitors on stone formation [25]. Xiao et al. performed a cross-sectional analysis in order to investigate this further amongst a population with type 2 diabetes mellitus (DM) [26]. The protection achieved among women was superior to men. This may be due to androgen-mediated calcium excretion in male kidneys counteracting the protection derived from ACE inhibitors.

### Stone Composition

Three studies were identified which investigated the association of stone composition and gender [27–29]. Kravdal et al. analysed a series of over 1250 stones collected at surgery from a Norwegian population [27]. They found that men were more likely to have calcium oxalate monohydrate stones than women (males, 57.3% vs females, 47.1%, *p* < 0.001), and women were more likely to have carbonate apatite (females, 16.2% vs males, 7.8%, *p* < 0.001) and struvite (females, 10.6% vs males, 3.0%, *p* < 0.001) stones. Wang et al. performed a similar analysis of over 1500 stones collected at their tertiary centre [28]. Women were more likely to have mixed composition stones (females, 70.9% vs males, 57.0%, *p* < 0.001) and more likely to have a history of urinary tract infection (UTI) (females, 39.1% vs males, 35.7%, *p* = 0.042). They also found that women were statistically more likely to have renal as opposed to ureteric stones (females, 85.2% vs males, 77.4%, *p* < 0.001).

Wood et al. published an analysis of over 500 stone patients who had 24-h urine collections performed [29]. This showed that men excreted more calcium (mg/day) (males, 217.7 vs females, 180.1, *p* = 0.0002) and oxalate (mg/day) (males, 43.2 vs females, 34.20, *p* < 0.0001) into their urine than women, had a lower pH than women (males, 6.03 vs females, 6.20, *p* = 0.0001) and were more likely to have supersaturation of uric acid in their urine than women (mg/day) (males, 1.03 vs females, 0.70, *p* < 0.0001). The latter is consistent with existing literature highlighting that women are less at risk of high urate levels than men, resulting in a male predominance in uric acid stones and gout [30]. The basis for this may be hormonal, and they found multiple mechanisms by which the expression and activity of uric acid transporters are affected by oestrogen, which goes some way to explain the gender difference in pathology related to hyperuricaemia.

### Post-intervention Sepsis

Sepsis is the leading cause of KSD-related mortality [31]. Seven studies were identified, which investigated sepsis post-KSD intervention [32–38]. It has previously been demonstrated that women have higher sepsis rates after ureteroscopy (URS) than men [39]. Southern et al. confirmed this in a retrospective analysis of over 3000 URS cases (OR 1.6, CI 1.19–2.15) [32]. Another study recorded a significantly higher rate of post-operative sepsis after percutaneous nephrolithotomy (PCNL) in women (OR 7.8, *p* = 0.001) [33]. Overall, all the studies determined female gender to be a risk factor for developing sepsis post-stone surgery [32–38].

### Gender Difference in Paediatrics

The prevalence of KSD is also increasing in the paediatric setting [40]. A large contributing factor for this is considered to be the paralleled rise of body mass index (BMI) in this age group [41]. Three studies were identified, which investigated gender differences in paediatric KSD [42–44]. Taisan et al. reported findings from a longitudinal study over a 30-year period and found the overall risk of KSD doubled in children [42]. Furthermore, the lifetime risk of developing KSD among females increased by 45% during the study period (1997–2012). Both this study and one by Schwadrerer et al. recorded that girls were most likely to experience KSD in adolescence [42, 43]. This age group is increasingly affected by KSD which is consistent with findings from NHNES [10]. Meiouiet et al. studied stone composition in 432 paediatric calculi [44]. Girls had calcium oxalate stones in 72.3% of cases compared to 42.7% in boys (*p* < 0.00001).

### KSD and Ambient Temperature

Three studies were identified, which investigated gender differences in the setting of warmer seasons [45, 47, 48]. Vicedo-Cabrera et al. analysed data from admission to emergency department (ED) across 68 centres in a US state between 1997 and 2015 [45]. When the highest ambient temperatures were recorded, a significantly higher proportion of men were admitted with KSD (males, RR 1.73 vs females, RR 1.15, *p* < 0.001). This is consistent with previous evidence highlighting the seasonal variation in KSD events as well as a recent population-based time series analysis in Canada (RR = 1.64 vs 1.22, *p* = 0.006) [46, 47]. Fukuhara et al. also reported that more ageing and male patients presented with acute stone disease when the ambient temperature was high [48]. It is difficult however to determine whether the cause is related to occupational behaviour or whether there may be a truly physiological cause. The latter may be because there are more aquaporin channels in the proximal collecting tubule and thin descending loop of Henle in men, which causes greater water reabsorption and therefore more concentrated urine [49]. This is consistent with a previous study which showed that men have a lower urine volume in hot weather than women [46].

### Limitations and Future Research

This review offers insight into the evolving changes in the global burden of KSD and the gender differences that exist within it (Table 2). The heterogeneity of studies precludes a formal meta-analysis. Furthermore, the nature of included studies introduces several limitations. Cause-effect conclusions are difficult to establish in cross-sectional studies and case control studies, such as where patients record stone events are subject to recall bias.

**Table 2:** Summary of findings on gender differences on different areas of interest

Theme	Summary
Gender gap	Rise in prevalence of KSD is greater among women than men
Stone composition	Men more likely than women to have metabolic disorders (obesity, type 2 diabetes mellitus) and present at a younger age than women Men excrete more calcium and oxalate into their urine than women, have a lower urine pH and are more likely to have uric acid supersaturation than women
Quality of Life	Women’s quality of life is more affected by KSD than men, with worse QoL in most domains
Role of hormones	Oestrogen produces a favourable renal environment to protect against nephrolithiasis, while oestrogen antagonists cause increased oxalate excretion and deposition
Post procedure sepsis	Women are more likely to develop sepsis after procedures such as ureteroscopy and percutaneous nephrolithotomy
Paediatrics	Prevalence of KSD is rising among children. In paediatric setting, females most likely to suffer KSD during adolescence
Ambient temperature	Men have a bigger increase in stone presentations during warm weather than their female counterparts

In addition to the abovementioned possible causes for the changes to the gender gap, the rise in obesity among women, which is greater than among men, is likely a contributing factor. Studies have shown obesity in women leads to significantly greater supersaturation of calcium oxalate [50]. Goldfarb et al. reported findings from a twin registry and revealed the heritable component of KSD to be greater in men than women [51]. Their findings support the theory that environmental risk factors play a greater role in women.

Data from the Nurses’ Health Study (NHS) I and II showed long-term (> 2 months) antibiotic usage is an independent risk factor for KSD [52]. Given the proportionally higher volume of women who require long-term antibiotics for chronic UTI, this may be another under-recognized contributor to the epidemiological changes observed. More needs to be done on standardizing the outcome parameters and evaluating the QoL of these patients [53].

## Conclusion

Recent literature demonstrates a temporal change in the disease burden of KSD among men and women. The latter, especially adolescent females, are more likely to develop KSD in their lifetime compared to previous eras. Determining causation is complex, and continued research is warranted. As our knowledge of risk factors for KSD deepens, it should be mirrored in the dissemination of patient education to help remedy these global trends. Clinicians should strive to deliver a personalised stone approach as much as possible.

## References

[1] Scales CD Jr, Smith AC, Hanley JM, Saigal CS. Urologic Diseases in America Project. Prevalence of kidney stones in the United States. Eur Urol. 2012;62(1):160–5. 10.1016/j.eururo.2012.03.052. Epub 2012 Mar 31. PMID: 22498635; PMCID: PMC3362665. 10.1016/j.eururo.2012.03.052 PMC3362665 22498635

[2] Eisner BH, Goldfarb DS. A nomogram for the prediction of kidney stone recurrence. J Am Soc Nephrol. 2014;25(12):2685–7. 10.1681/ASN.2014060631. Epub 2014 Aug 7. PMID: 25104802; PMCID: PMC4243365. 10.1681/ASN.2014060631 PMC4243365 25104802

[3] Antonelli JA, Maalouf NM, Pearle MS, Lotan Y (2014). Use of the national health and nutrition examination survey to calculate the impact of obesity and diabetes on cost and prevalence of urolithiasis in 2030. Eur Urol.

[4] Saigal CS, Joyce G, Timilsina AR. Urologic Diseases in America Project. Direct and indirect costs of nephrolithiasis in an employed population: opportunity for disease management? Kidney Int. 2005;68(4):1808–14. 10.1111/j.1523-1755.2005.00599.x. PMID: 16164658. 10.1111/j.1523-1755.2005.00599.x 16164658

[5] Geraghty RM, Cook P, Walker V, Somani BK (2020). Evaluation of the economic burden of kidney stone disease in the UK: a retrospective cohort study with a mean follow-up of 19 years. BJU Int.

[6] Jones P, Karim SS, Gamage KN et al. Do lifestyle factors including smoking, alcohol, and exercise impact your risk of developing kidney stone disease? Outcomes of a systematic review. J Endourol. 2020; 10.1089/end.2020.0378. Epub ahead of print. PMID: 32808537. 10.1089/end.2020.0378 32808537

[7] Ping H, Lu N, Wang M, Lu J, Liu Y, Qiao L, Wang Y, Jiang L, Zhang X (2019). New-onset metabolic risk factors and the incidence of kidney stones: a prospective cohort study. BJU Int.

[8] Andreassen KH, Pedersen KV, Osther SS, Jung HU, Lildal SK, Osther PJ (2016). How should patients with cystine stone disease be evaluated and treated in the twenty-first century?. Urolithiasis.

[9] Preferred reporting items for systematic review and meta-analysis protocols (PRISMA-P). elaboration and explanation. BMJ. 2016;21;354:i4086. 10.1136/bmj.i4086. Erratum for: BMJ. 2015 Jan 02;02; 02;350:g7647. PMID: 27444514. 10.1136/bmj.g7647 25555855

[10] Abufaraj M, Xu T, Cao C, Waldhoer T, Seitz C, D'andrea D, Siyam A, Tarawneh R, Fajkovic H, Schernhammer E, Yang L, Shariat SF. Prevalence and trends in kidney stone among adults in the USA: analyses of National Health and Nutrition Examination Survey 2007–2018 Data. Eur Urol Focus. 2020;S2405–4569(20)30224–8. 10.1016/j.euf.2020.08.011. Epub ahead of print. PMID: 32900675. 10.1016/j.euf.2020.08.011 32900675

[11] Tundo G, Khaleel S, Pais VM (2018). Gender equivalence in the prevalence of nephrolithiasis among adults younger than 50 years in the United States. J Urol.

[12] Chen Z, Prosperi M, Bird VY (2019). Prevalence of kidney stones in the USA: the National Health and Nutrition Evaluation Survey. Journal of Clinical Urology.

[13] Hsi RS, Kabagambe EK, Shu X, Han X, Miller NL, Lipworth L. Race- and sex-related differences in nephrolithiasis risk among blacks and whites in the southern community cohort study. Urology. 2018;118:36–42. 10.1016/j.urology.2018.04.036. Epub 2018 May 10. PMID: 29753847; PMCID: PMC7050473. 10.1016/j.urology.2018.04.036 PMC7050473 29753847

[14] Masterson JH, Phillips CJ, Crum-Cianflone NF, Krause RJ, Sur RL, L'Esperance JO (2017). A 10-year retrospective review of nephrolithiasis in the navy and navy pilots. J Urol.

[15] Kittanamongkolchai W, Vaughan LE, Enders FT, Dhondup T, Mehta RA, Krambeck AE, McCollough CH, Vrtiska TJ, Lieske JC, Rule AD. The changing incidence and presentation of urinary stones over 3 decades. Mayo Clin Proc. 2018;93(3):291–299. 10.1016/j.mayocp.2017.11.018. Epub 2018 Feb 14. PMID: 29452705; PMCID: PMC5849397. 10.1016/j.mayocp.2017.11.018 PMC5849397 29452705

[16] Vaughan LE, Enders FT, Lieske JC, Pais VM, Rivera ME, Mehta RA, Vrtiska TJ, Rule AD. Predictors of symptomatic kidney stone recurrence after the first and subsequent episodes. Mayo Clin Proc. 2019;94(2):202–210. 10.1016/j.mayocp.2018.09.016. Epub 2018 Dec 4. PMID: 30527866; PMCID: PMC6390834. 10.1016/j.mayocp.2018.09.016 PMC6390834 30527866

[17] Mehmi A, Jones P, Somani BK. Current status and role of Patient-reported Outcome Measures (PROMs) in endourology. Urology. 2020; 28:S0090–4295(20)31130–4. 10.1016/j.urology.2020.09.022. Epub ahead of print. PMID: 32991909 10.1016/j.urology.2020.09.022 32991909

[18] Stern KL, Gao T, Antonelli JA, Viprakasit DP, Averch TD, Chi T, Chew BH, Bird VG, Pais VM, Streeper NM, Sur RL, Nakada SY, Penniston KL, Sivalingam S (2019). Association of patient age and gender with kidney stone related quality of life. J Urol.

[19] MacCraith E, O’Kelly J, Ryan J (2020). Predictors of emergency department attendance following ureterorenoscopy for urolithiasis. Ir J Med Sci.

[20] Patel N, Brown RD, Sarkissian C, De S, Monga M. Quality of life and urolithiasis: the patient - reported outcomes measurement information system (PROMIS). Int Braz J Urol. 2017;43(5):880–886. 10.1590/S1677-5538.IBJU.2016.0649. PMID: 28792186; PMCID: PMC5678519. 10.1590/S1677-5538.IBJU.2016.0649 PMC5678519 28792186

[21] Islamoglu E, Tas S, Karamik K, Yalcinkaya S, Tokgoz H, Savas M (2019). Does extracorporeal shock wave lithotripsy-related pain get affected by menstrual cycle and menopause?. Urolithiasis.

[22] Peerapen P, Thongboonkerd V. Protective cellular mechanism of estrogen against kidney stone formation: a proteomics approach and functional validation. proteomics. 2019;19(19):e1900095. 10.1002/pmic.201900095. Epub 2019 Sep 18. PMID: 31475403. 10.1002/pmic.201900095 31475403

[23] Zhu W, Zhao Z, Chou FJ, Zuo L, Liu T, Bushinsky D, Chang C, Zeng G, Yeh S (2019). The protective roles of estrogen receptor *β* in renal calcium oxalate crystal formation *via* reducing the liver oxalate biosynthesis and renal oxidative stress-mediated cell injury. Oxid Med Cell Longev.

[24] Nackeeran S, Katz J, Ramasamy R (2020). Association between sex hormones and kidney stones: analysis of the National Health and Nutrition Examination Survey. World J Urol.

[25] Rathod A, Bonny O, Guessous I, Suter PM, Conen D, Erne P (2015). Association of urinary calcium excretion with serum calcium and vitamin D levels. Clin J Am Soc Nephrol.

[26] Xiao Y, Wei L, Xiong X, Yang Y, Li L, Yang M, Deng F, Sun L. Sex differences in kidney stone disease in Chinese patients with type 2 diabetes mellitus. Kidney Dis (Basel). 2020;6(3):195–203. 10.1159/000506053. Epub 2020 Feb 28. PMID: 32523961; PMCID: PMC7265720. 10.1159/000506053 PMC7265720 32523961

[27] Kravdal G, Helgø D, Moe MK (2019). Kidney stone compositions and frequencies in a Norwegian population. Scandinavian Journal of Urology.

[28] Wang S, Zhang Y, Zhang X, Tang Y, Li J. Upper urinary tract stone compositions: the role of age and gender. Int Braz J Urol. 2020;46(1):70–80. 10.1590/S1677-5538.IBJU.2019.0278. PMID: 31851461; PMCID: PMC6968895. 10.1590/S1677-5538.IBJU.2019.0278 PMC6968895 31851461

[29] Wood K, Boyd C, Whitaker D, Ashorobi O, Poore W, Gower B, Assimos DG. Impact of Demographic factors and systemic disease on urinary stone risk parameters amongst stone formers. Rev Urol. 2019;21(4):158–165. PMID: 32071564; PMCID: PMC7020277. PMC7020277 32071564

[30] Halperin Kuhns VL, Woodward OM (2020). Sex differences in urate handling. Int J Mol Sci.

[31] Whitehurst L, Jones P, Somani BK (2019). Mortality from kidney stone disease (KSD) as reported in the literature over the last two decades: a systematic review. World J Urol.

[32] Southern JB, Higgins AM, Young AJ, Kost KA, Schreiter BR, Clifton M, Fulmer BR, Garg T (2019). Risk factors for postoperative fever and systemic inflammatory response syndrome after ureteroscopy for stone disease. J Endourol.

[33] Lorenzo Soriano L, Ordaz Jurado DG, Pérez Ardavín J, Budía Alba A, Bahílo Mateu P, Trassierra Villa M, López Acón D. Predictive factors of infectious complications in the postoperative of percutaneous nephrolithotomy. Actas Urol Esp. 2019;43(3):131–136. English, Spanish. 10.1016/j.acuro.2018.05.009. Epub 2018 Nov 8. PMID: 30415829. 10.1016/j.acuro.2018.05.009 30415829

[34] Nevo A, Mano R, Baniel J, Lifshitz DA (2017). Ureteric stent dwelling time: a risk factor for postureteroscopy sepsis. BJU Int.

[35] Díaz Pérez D, Laso García I, Sánchez Guerrero C, Fernández Alcalde Á, Ruiz Hernández M, Brasero Burgos J, Lorca Álvaro J, Duque Ruiz G, Arias Funez F, Burgos Revilla FJ. Urinary sepsis after endourological ureterorenoscopy for the treatment of lithiasis. Actas Urol Esp. 2019;43(6):293–299. English, Spanish. 10.1016/j.acuro.2019.02.001. Epub 2019 May 2. PMID: 31056221. 10.1016/j.acuro.2019.02.001 31056221

[36] Baboudjian M, Gondran-Tellier B, Abdallah R, Sichez PC, Akiki A, Gaillet S, Delaporte V, Karsenty G, Lechevallier E, Boissier R (2020). Predictive risk factors of urinary tract infection following flexible ureteroscopy despite preoperative precautions to avoid infectious complications. World J Urol.

[37] Özsoy M, Acar Ö, Sarica K (2015). Impact of gender on success and complication rates after ureteroscopy. World J Urol.

[38] Wood B, Habashy D, Mayne DJ, Dhar A, Purvis C, Skyring T (2020). The utility of preoperative and intraoperative cultures for guiding urosepsis empirical treatment. Journal of Clinical Urology.

[39] Chugh S, Pietropaolo A, Montanari E (2020). Predictors of urinary infections and urosepsis after ureteroscopy for stone disease: a systematic review from EAU Section of Urolithiasis (EULIS). Curr Urol Rep.

[40] Pietropaolo A, Proietti S, Jones P, Rangarajan K, Aboumarzouk O, Giusti G, Somani BK (2017). Trends of intervention for paediatric stone disease over the last two decades (2000–2015): a systematic review of literature. Arab J Urol.

[41] Alfandary H, Haskin O, Davidovits M, Pleniceanu O, Leiba A, Dagan A (2018). Increasing prevalence of nephrolithiasis in association with increased body mass index in children: a population based study. J Urol.

[42] Tasian GE, Ross ME, Song L, Sas DJ, Keren R, Denburg MR, Chu DI, Copelovitch L, Saigal CS, Furth SL. Annual incidence of nephrolithiasis among children and adults in South Carolina from 1997 to 2012. Clin J Am Soc Nephrol. 2016;11(3):488–96. 10.2215/CJN.07610715. Epub 2016 Jan 14. PMID: 26769765; PMCID: PMC4791823. 10.2215/CJN.07610715 PMC4791823 26769765

[43] Schwaderer AL, Raina R, Khare A, Safadi F, Moe SM, Kusumi K (2019). Comparison of risk factors for pediatric kidney stone formation: the effects of sex. Front Pediatr.

[44] Meiouet F, El Kabbaj S, Daudon M (2019). Pediatric urolithiasis in Morocco: composition of 432 urinary calculi analyzed by infrared spectroscopy. Prog Urol.

[45] Vicedo-Cabrera AM, Goldfarb DS, Kopp RE, Song L, Tasian GE. Sex differences in the temperature dependence of kidney stone presentations: a population-based aggregated case-crossover study. Urolithiasis. 2020;48(1):37–46. 10.1007/s00240-019-01129-x. Epub 2019 Mar 21. PMID: 30900001; PMCID: PMC7357996. 10.1007/s00240-019-01129-x PMC7357996 30900001

[46] Parks JH, Barsky R, Coe FL (2003). Gender differences in seasonal variation of urine stone risk factors. J Urol.

[47] Ordon M, Welk B, Li Q, Wang J, Lavigne E, Yagouti A, Copes R, Cakmak S, Chen H (2016). Ambient temperature and the risk of renal colic: a population-based study of the impact of demographics and comorbidity. J Endourol.

[48] Fukuhara H, Ichiyanagi O, Kakizaki H, Naito S, Tsuchiya N. Clinical relevance of seasonal changes in the prevalence of ureterolithiasis in the diagnosis of renal colic. Urolithiasis. 2016;44(6):529–537. 10.1007/s00240-016-0896-3. Epub 2016 Jun 17. PMID: 27314408; PMCID: PMC5063892. 10.1007/s00240-016-0896-3 PMC5063892 27314408

[49] Brikowski TH, Lotan Y, Pearle MS. Climate-related increase in the prevalence of urolithiasis in the United States. Proc Natl Acad Sci U S A. 2008;105(28):9841–6. 10.1073/pnas.0709652105. Epub 2008 Jul 14. PMID: 18626008; PMCID: PMC2474527. 10.1073/pnas.0709652105 PMC2474527 18626008

[50] Tavasoli S, Taheri M, Khoshdel A, Basiri A (2017). Association of body mass index, waist circumference, and waist-stature ratio with urine composition in patients with urolithiasis. Iran J Kidney Dis.

[51] Goldfarb DS, Avery AR, Beara-Lasic L, Duncan GE, Goldberg J (2018). A twin study of genetic influences on nephrolithiasis in women and men. Kidney Int Rep.

[52] Ferraro PM, Curhan GC, Gambaro G, Taylor EN. Antibiotic use and risk of incident kidney stones in female nurses. Am J Kidney Dis. 2019;74(6):736–741. 10.1053/j.ajkd.2019.06.005. Epub 2019 Sep 19. PMID: 31543288; PMCID: PMC7006481. 10.1053/j.ajkd.2019.06.005 PMC7006481 31543288

[53] New F, Somani BK (2016). A complete world literature review of Quality oof Life (QoL) in patients with kidney stone disease (KSD). Curr Urol Rep.

